# Parenting Behavior in Mothers of Preschool Children with ASD: Development of a Self-Report Questionnaire

**DOI:** 10.1155/2015/381236

**Published:** 2015-10-29

**Authors:** Greet Lambrechts, Jarymke Maljaars, Hannah Boonen, Lotte van Esch, Karla Van Leeuwen, Ilse Noens

**Affiliations:** ^1^Parenting and Special Education Research Unit, University of Leuven (KU Leuven), Leopold Vanderkelenstraat 32, P.O. Box 3765, 3000 Leuven, Belgium; ^2^Leuven Autism Research (LAuRes), University of Leuven (KU Leuven), 3000 Leuven, Belgium

## Abstract

Parents of young children with autism spectrum disorder (ASD) encounter many daily challenges and often experience much stress. However, little research exists about parenting behavior among these parents. With this study, we aim to address this gap. We examined the structure and internal consistency of a questionnaire intended to measure parenting behavior among mothers of young children with ASD. Furthermore, we compared parenting behavior among mothers of young children with and without ASD between two and six years old. Factor analyses resulted in a factor solution with seven subscales of parenting behavior. Two additional subscales especially relevant for parenting preschoolers with ASD were also considered. Analyses of covariance, controlling for gender and age, showed significantly higher scores for *Discipline* and *Stimulating the Development* in the control group in comparison with the ASD group. These findings suggest that mothers of preschoolers with ASD are still trying to find strategies to guide and stimulate their child's behavior and development effectively.

## 1. Introduction

Parents of children with autism spectrum disorder (ASD) encounter many challenges on a daily basis. A great number of studies refer to higher levels of stress among parents of children with ASD when compared to parents of typically developing children or parents of children with other disabilities (e.g., [1–5]). Whether parents experience stress or not depends on their coping strategies, informal social support sources, beliefs about the efficacy of the interventions, and the level of autism symptomatology [3]. Apart from the daily stress of raising a child with ASD, parents of young children (between two and six years old) also have to cope with the emotions of a recent ASD diagnosis and the initiation of intervention services [6]. Furthermore, during this period, young children are likely to show many “early autism” deficits, such as a lack of social responsiveness, communication skills, joint attention, and interactive play skills. These deficits can cause considerable concern for parents who may question their own parenting abilities as a result [7]. Literature suggests that parents of young children with ASD who are emotionally unable to accept their family's situation may have more difficulties attuning to their children's needs [8].

Despite the evidence that parenting young children with ASD is rather challenging, little research exists about parenting behavior among parents of young children with ASD as such. Previous research has primarily investigated characteristics of the parent-child relationship, such as emotional availability and attachment in families with a young child with ASD [9]. van Ijzendoorn et al. [9], for example, studied sensitivity and attachment in parents of children with ASD using the strange situation procedure [10] and the Emotional Availability Scales (EAS Infancy/Early Childhood Version; [11]). However, in the present study, parenting behavior is rather considered as the observable behavior of parents which plays a role in the socialization of children or the way in which children acquire the social, emotional, and cognitive skills to function in the social community [12]. Literature about typically developing children generally distinguishes between two main dimensions: parental warmth (support or responsiveness) and parental control (behavioral and psychological control) [13]. Maccoby and Martin [13] identified four patterns of parenting based on these two dimensions: authoritarian, authoritative, permissive, and indifferent parenting. More recently, growing attention is being given to autonomy support in addition to these two dimensions [14]. Examples of autonomy support are showing empathy for the perspective of the child, giving choices, and encouraging the child to act on the basis of intrinsic satisfying motives [15].

Only a few studies have examined these dimensions of parenting or closely related variables in parents of young children with ASD. First, Kasari et al. [16] compared the caregiver's (mother or father) responsiveness in three groups of preschool children (a group with ASD, a group with a developmental disability, and a control group without a disability), but they did not observe any significant differences between the groups. Second, in a study by Watson [17], mothers of preschool children with ASD showed as much responsiveness as mothers of typically developing preschool children. And, finally, Baker et al. [18] did not find differences in sensitivity between mothers of toddlers with emergent ASD and mothers of toddlers without an eventual ASD diagnosis.

As regards the parental control dimension, Kasari et al. [16] showed that caregivers of children with ASD and children with a developmental disability used more control strategies than caregivers of the control group. Similar results were found in a study by Lemanek et al. [19] of preschoolers with ASD and in a study by Wan et al. [20] of infants at risk for ASD.

Some studies focused on specific aspects of parenting behavior. Meirsschaut et al. [21] concluded that mothers of preschoolers with ASD used more social and imperative initiatives, less declarative initiatives, and more denying responses towards their child in comparison to mothers of typically developing children. Furthermore, mothers of preschoolers with ASD used as many social initiatives and reacted as responsively with an unfamiliar child as with their own child. Blacher et al. [22] examined positive and negative parenting behaviors among parents of children with developmental delays (i.e., ASD, Down syndrome, and cerebral palsy) in comparison to parents of typically developing children at three, four, and five years of age. Parents of children with developmental delays showed more negative parenting behavior (i.e., negative affect, intrusiveness, and detachment) in comparison with the control group at the three time points. Parents of the clinical group showed less positive parenting behavior (i.e., positive affect, sensitivity, and stimulation of cognition) in comparison with the control group but only when the child was three years old.

Finally, Rutgers et al. [23] focused specifically on parenting styles instead of parenting dimensions and they compared parenting styles among parents of preschoolers with ASD with parenting styles among parents of children of the same age with an intellectual disability, with a language disorder, and without a diagnosis. Parents of typically developing children reported higher levels of authoritative parenting (a combination of high levels of demandingness and responsiveness) in comparison with all other clinical groups.

In summary, the majority of studies reported no differences between the clinical groups and the control group in terms of responsiveness. Conversely, regarding control strategies, differences were often observed, with higher scores in the clinical groups. However, results across studies are inconclusive, thus highlighting the need for more research. Furthermore, the clinical groups often consist of a combination of parents of children with ASD and parents of children with another developmental disorder. This makes it difficult to draw conclusions about parents of children with ASD specifically.

Except for the study by Rutgers et al. [23], all these studies used observations to study parenting behavior. Although observations have the advantage of presenting a more “objective” view on parenting, self-report measures on parenting can also provide useful information: they give insight into the parents' point of view about behavior in different daily situations. Moreover, an instrument that could prompt parents to have a conversation about their parenting behavior would be valuable, particularly in clinical applications. In the study by Rutgers et al. [23], parents completed a questionnaire assessing authoritative and authoritarian child-rearing styles. Osborne and Reed [5] presented the parents of children with ASD between 2 and 16 years old with a self-report instrument with four parenting subscales, involvement, communication, limit setting, and autonomy, in combination with a parental stress measure. In addition to these already available questionnaires, it would be interesting to have a self-report instrument to measure a broad range of general parenting behaviors combined with a focus on parenting behavior specifically relevant for young children with ASD. In the current study, we aimed at evaluating such a self-report questionnaire of parenting behavior and characterizing parenting behavior among mothers of young children with ASD.

In a preliminary questionnaire study about parenting behavior among parents of children with ASD between 8 and 18 years old [24], we found, apart from the general parenting behaviors, indications for parenting behaviors specifically relevant for children with ASD. We developed an instrument similar to this earlier questionnaire for children younger than 6 years old based on the Parental Behavior Scale for toddlers (PBS-T; [25]).

First, we examined the factor structure and internal consistency of this adjusted questionnaire to measure parenting behavior among mothers of young children with ASD. Second, we compared parenting behavior among mothers of young children with and without ASD. Based on earlier research, we did not expect to find any differences on the subscale Positive Parenting between the two groups but more behavioral control and behaviors specifically relevant for parenting children with ASD in the ASD group.

## 2. Materials and Methods

### 2.1. Participants and Procedure

A total of 1314 Dutch-speaking mothers (Flemish part of Belgium and Netherlands) participated in this study. The ASD group consisted of 57 biological mothers of a child with ASD (*n* = 37 from Netherlands and *n* = 20 from the Flemish part of Belgium). All children were between 31 months and 72 months old (*M* = 55.37; SD = 11.21) and 84.2% (*n* = 48) were boys. Ten children had an intellectual disability, 8 children had an IQ below average (between 70 and 84), 20 children had an average IQ (between 85 and 115), and 6 children had an IQ above average (higher than 115). For 13 children, there was no information available about their cognitive functioning. Mothers indicated on a questionnaire that their child had received a clinical ASD diagnosis. The control group consisted of 1257 biological mothers with a child without ASD (*n* = 138 from Netherlands and *n* = 1119 from the Flemish part of Belgium). All children were between 20 and 73 months old (*M* = 36.93; SD = 9.72) and 52.3% (*n* = 658) were boys. For the factor analyses, data of the entire control group were used, while the group comparisons were based on the data of a subgroup matched with the ASD group.

All mothers participated in the context of several research projects on parenting behavior and/or ASD in young children. After informed consent was given, they received the questionnaire by mail or during a home visit. The majority of the mothers of the control group (*n* = 882) filled out the Parental Behavior Scale for toddlers (PBS-T; [22]). All the other mothers filled out the adjusted version of the measure supplemented with additional items tapping parenting behaviors hypothesized to be specifically relevant in the context of ASD. The research design was approved by the university's ethical committee and mothers were not reimbursed for participating.

### 2.2. Measure

The Parental Behavior Scale for toddlers (PBS-T; [25]) is designed to measure general parenting behavior among parents of children younger than four years old. This questionnaire is based on the Parental Behavior Scale (PBS; [26]) for parents of school-aged children. For the purpose of studying parenting in families with school-aged children with ASD, this PBS version for parents of school-aged children has been extended with items tapping parenting behaviors specifically relevant in the context of ASD, such as adapting the child's environment and stimulating the child's development (PBS-A; [27]). In line with that extension, we also adjusted the Parental Behavior Scale for toddlers and added several items. First of all, we selected all the items of this scale about general parenting behavior that were applicable to children between two and six years old (*n* = 40):* Positive Parenting* (e.g., I play with my child),* Autonomy Support* (e.g., I give my child the opportunity to try new things),* Supervision and Safety* (e.g., I provide a safe environment for my child),* Discipline* (e.g., when my child does something I do not like, I raise my voice),* Rules and Structure* (e.g., I make sure my child's daily routine is the same day in, day out), and* Rewarding* (e.g., when my child masters doing something new, I applaud or say good boy/good girl). Furthermore, 21 items specifically relevant for parenting children with ASD were added about* Stimulating the Development* (e.g., when a person is crying, I explain to my child how this person is feeling and why) and* Adapting the Environment* (e.g., when my child is doing an activity (e.g., drawing or reading a book), I keep distraction to a minimum). This self-report measure is to be completed by parents of children up to six years old. By means of a 5-point Likert scale, parents rate the frequency of each behavior (1 = (almost) never, 2 = little, 3 = sometimes, 4 = often, and 5 = (almost) always).

### 2.3. Data Analysis

All statistical tests were performed using IBM SPSS Statistics 22. Because the ASD group is much smaller than the control group, we decided to carry out the factor analyses only on the data of the control group that consisted of 1257 biological mothers of a child without ASD. First, we checked the assumptions to execute a Principal Axis Factoring (PAF). The Kaiser-Meyer-Olkin (KMO) value was greater than .8 and Bartlett's test of sphericity was significant (*p* < .001) indicating that we could execute a PAF [28]. We chose to use a direct oblimin rotation, because we expected the factors to be correlated.

To conduct the comparative analyses, we created a matching control group by randomly selecting respondents from the control group, based on the criteria of child age (*M* = 55.6 months in the control group and *M* = 55.4 in the ASD group) and child sex (9 girls and 48 boys in both groups). We used a MANCOVA with group and sex as factors, age as a covariate, and mean scores for the parenting subscales as dependent variables. The error variance was equal across groups for most of the subscales (Levene's tests, *p* > .05), except for* Supervision and Safety* and for* Stimulating the Development*. To address the violation of homogeneity for the latter subscales, we checked the Brown-Forsythe* F* and Welch* F* statistics of the One-Way ANOVA analysis of these subscales. Eta squared values were used as indicators of effect size with values of .01–.06 pointing to a small effect, .06–.14 pointing to a medium effect, and >.14 pointing to a large effect [29].

## 3. Results

### 3.1. Factor Structure of the General Parenting Items

The Kaiser criterion (select all factors with eigenvalue > 1) suggested a ten-factor solution with many factors explaining only little variance and/or comprising just a few items. However, the drop in the scree plot suggested a seven-factor solution [30] and because this solution is also more acceptable from a conceptual point of view we decided to continue with seven factors. This solution accounted for 34.10% of the variance. We included items only if they loaded higher than |.35| on one factor in the pattern matrix and lower than |.30| on the other factors. This resulted in a* Positive Parenting* subscale of 6 items, an* Autonomy Support* subscale of 4 items, a* Rules and Structure* subscale of 3 items, a* Discipline* subscale of 5 items, a* Rewarding* subscale of 3 items, a* Supervision and Safety* subscale of 6 items, and an* Indulging* subscale of 2 items (e.g., when my child wants something, I give in so he or she will not get cross or start to cry). Eleven items were removed.

The internal consistency was good for five subscales (.70 < *α* < .81; corrected item total correlations >.36). For the* Discipline* subscale, the internal consistency was rather poor, with Cronbach's alpha value of .57 (corrected item total correlations >.25). Pearson's correlation between the two items of the* Indulging* subscale was high (*r* = .48; *p* < .001). *α* values and the intercorrelations of the seven factors are presented in Table 1.

### 3.2. Items Specifically Relevant for Parenting Preschoolers with ASD

Although it is advisable to conduct a factor analysis on the ASD-related items as well, the ASD group was too small to obtain meaningful results. Therefore we decided to use the original 21 items belonging to the two subscales based on the solution in the study by Lambrechts et al. [24]:* Stimulating the Development* (*n* = 13 items) and* Adapting the Environment* (*n* = 8 items).

The calculation of the internal consistency was based on the data of all mothers who filled in the adjusted version of the measure (*n* = 432). The internal consistency was good for* Stimulating the Development* subscale (*α* = .81; corrected item total correlations >.22) and rather weak for* Adapting the Environment* subscale (*α* = .56; corrected item total correlations >.14).

### 3.3. Comparing Parenting Behavior between the Control and the ASD Group

Significant main effects of the factor “group” were present for the subscales* Discipline* (*F*_(1,109)_ = 13.22; *p* < .001; *η* ^2^ = .10) and* Stimulating the Development* (Brown-Forsythe *F*_(1,99.495)_ = 14.14, *p* < .001, and Welch *F*_(1,99.495)_ = 14.14, *p* < .001), with higher mean scores for the control group in comparison with the ASD group.  Table 2 shows the main results of these analyses. For* Supervision and Safety*, Brown-Forsythe* F* and Welch* F* statistics showed the same result as* F*-test.

Regarding the age of the children, MANCOVA showed a significant main effect for the* Discipline* subscale (*F*_(1,109)_ = 7.75;  *p* < .01; *η* ^2^ = .06). When checking Spearman's*ρ* correlations, a significant correlation was found between age and* Discipline* subscale in the control group only (*ρ* = −.27, *p* < .05 for the control group; *ρ* = −.23, *p* = .09 for the ASD group), with parents of older children reporting using less discipline.

## 4. Discussion

In this study we examined the factor structure and internal consistency of a new questionnaire to measure parenting behavior among mothers of young children with ASD. With regard to the general parenting items, all the theoretically expected scales were found, with one extra subscale referring to indulging behavior. All but one subscale yielded good internal consistency;* Discipline* subscale should be considered with caution. Regarding the items specifically relevant for parenting preschoolers with ASD, further analyses of a larger group of parents of a child with ASD are needed. For now, the original subscales, based on an instrument used in a previous study on parenting behavior among parents of older children with ASD [24], showed a good internal consistency score for* Stimulating the Development* subscale and a poorer internal consistency score for* Adapting the Environment* subscale.

With respect to the comparison between the control group and the ASD group, no differences were present for* Positive Parenting*. That was also the case for the other subscales that are related to Positive Parenting (*Autonomy Support*,* Rewarding*, and* Supervision and Safety*). These results are similar to the conclusions of other studies based on observational measurements [16–18].

For one aspect of* behavioral control* we found a significant difference between both groups with higher scores for* Discipline* in the control group. However, one should take the lower internal consistency score of this subscale into account. For other aspects of behavioral control (*Rules and Structure* and* Indulging*), there were no significant differences. Conversely, other studies revealed mostly higher scores for behavioral control in parents of children with or at risk for ASD [16, 19, 20]. A possible explanation for this contrast with previous research could be that in our study a distinction was made between several aspects of behavioral control and thus behavioral control was not treated as a general concept. Disciplining is a very specific act and only one aspect of behavioral control. It might be that mothers of young children with ASD interpret undesirable/unwanted behavior of their child differently. Often these behaviors cannot be labeled as unwillingness or deliberate misbehavior, and parents might be more inclined to search for preceding events that might provide insight into the behavior of their child [31].

Another finding regarding* Discipline* subscale revealed that disciplinary behaviors decreased with increasing age of the children in the control group. This effect was not present in the ASD group. Typically, young children's gains in representation, language, and self-concept support their emotional development. Consequently, intense emotional outbursts become less frequent over the preschool years [32] and this is possibly accompanied by less use of disciplining strategies when parenting older children. Young children with ASD, however, often have difficulties with representation, language, and self-concept [33, 34] and their emotional development may be slower and/or different, which can result in a different pattern of disciplining.

With respect to behaviors specifically relevant for parenting preschoolers with ASD, no group differences were found in* Adapting the Environment* subscale. Several items of this subscale are closely related to the general subscale* Rules and Structure* (e.g., “I make sure that everything has a definite place in our house” or “when my child is doing a certain activity, I limit possible distraction to a minimum”). Also for* Rules and Structure* subscale we did not find significant group differences. Given the age of the children involved in this study, this is perhaps not surprising. Almost all mothers of young children seem to provide structure. However, the mothers of the control group reported* Stimulating the Development* of their child more than the ASD group. One explanation could be that mothers of young children with ASD do not know exactly how to stimulate the development of their child and are still experimenting with ways to promote their children's psychosocial maturation. Additionally, some of the parenting behaviors included in* Stimulating the Development* subscale may not apply to young children with ASD (e.g., “when a person is crying, I explain to my child how this person is feeling and why” or “I stimulate pretend play”). However, the answers of parents to these items could be a good starting point for a conversation with parents about their needs in this context. Maybe they need more knowledge about parenting young children with ASD and/or maybe they need specific advice on how to stimulate their child's behavior in everyday situations.

Further research is needed to confirm these results. Concerning the subscales* Stimulating the Development* and* Adapting the Environment*, it may be interesting to look for items that are more specifically relevant for preschoolers with ASD.

The current results have to be interpreted with caution. First, for most children we obtained a confirmation of their ASD diagnosis but for some children we could only rely on their mothers' statement. Second, the two matched groups were rather small, and we could not perform a multigroup confirmatory factor analysis to check whether the factor structure of the questionnaire is comparable across groups of mothers with a child with ASD and mothers with a typically developing child. In the future, data of a different and lager group of both mothers and fathers with a child with ASD are needed to evaluate the scale properly.

Further research on the relationship with other children or parent variables and with observations of parenting behavior would be very interesting. As we mentioned in Introduction, many parents of preschoolers with ASD experience high levels of stress [3]. It would be interesting to study the relationship between parenting behavior and parental stress. Furthermore, it might be important to include a measure of the parents' social responsiveness because of the heritability of the disorder, which may influence their parenting behavior. Amongst other child variables, problem behavior and communication problems can play a significant role. Children with ASD, including very young children, have a relatively high chance of developing problem behavior [35] and parenting behavior can function as a risk factor as well as a protective factor in this area [26]. Additionally, communication problems are often related to problem behavior in children with ASD [36, 37].

Finally, after meeting the limitations mentioned before and doing more analyses on data from a larger ASD group, this instrument could be useful in practice. We think that it is important to have an instrument to screen parenting behavior among parents of young children with ASD in different domains. In contrast with observations of parenting behavior, a self-report questionnaire gives insight into the perspective of the parents concerning a variety of behaviors across a wide range of situations in a time efficient way. As we mentioned earlier, this can be a useful starting point for a conversation about parenting and for planning intervention in that context. Furthermore, this kind of instrument can also be used to evaluate interventions which focused on parenting behavior and to evaluate parenting as a moderator or mediator of treatment.

## Figures and Tables

**Table 1:** Number of items and *α* values for all the factors and their intercorrelations (Pearson's correlations).

Factors	Number of items	*α*	(2)	(3)	(4)	(5)	(6)	(7)
(1) Positive Parenting	6	.72	.43^*∗∗∗*^	.17^*∗∗∗*^	.01	.35^*∗∗∗*^	−.01	.34^*∗∗∗*^
(2) Autonomy Support	4	.79		.15^*∗∗∗*^	.06^*∗*^	.27^*∗∗∗*^	−.12^*∗∗∗*^	.24^*∗∗∗*^
(3) Rules and Structure	3	.75			.00	.14^*∗∗∗*^	−.14^*∗∗∗*^	.22^*∗∗∗*^
(4) Discipline	5	.57				.07^*∗*^	.00	−.05
(5) Rewarding	3	.81					−.05	.36^*∗∗∗*^
(6) Indulging	2	—						−.01
(7) Supervision and Safety	6	.71						

^*∗*^ *p* < .05; ^*∗∗∗*^ *p* < .001.

**Table 2:** Group differences in parenting behavior between the ASD and control group.

	Control	ASD	MANCOVA: ASD versus control		
	(*n* = 57) M (SD)	(*n* = 57) M (SD)	*F*	*p*	*η* ^2^
General parenting behavior					
Positive Parenting	4.02 (.40)	3.86 (.41)	3.55	.062	—
Autonomy Support	4.23 (.38)	4.00 (.40)	2.38	.126	—
Rules and Structure	4.41 (.49)	4.29 (.58)	0.03	.864	—
Discipline	**3.26 (.42)**	**2.93 (.51)**	**13.22**	**<.001**	.101^a^
Rewarding	4.61 (.41)	4.71 (.40)	1.29	.259	—
Indulging	2.19 (.56)	2.61 (.66)	2.31	.132	—
Supervision and Safety	4.22 (.38)	4.24 (.46)	0.16	.695	—
ASD-adapted parenting behavior					
Stimulating the Development	**3.96 (.38)**	**3.63 (.55)**	**5.11**	**.026**	.044^b^
Adapting the Environment	3.68 (.37)	3.73 (.49)	0.18	.672	—

^a^Medium effect size; ^b^small effect size.

## References

[1] Bristol M. M., Schopler E., Blacher J. (1984). A development perspective on stress and coping in families of autistic children. *Severely Handicapped Children and their Families*.

[2] Dumas J. E., Wolf L. C., Fisman S. N., Culligan A. (1991). Parenting stress, child behavior problems, and dysphoria in parents of children with autism, Down syndrome, behavior disorders, and normal development. *Exceptionality*.

[3] Hastings R. P., Johnson E. (2001). Stress in UK families conducting intensive home-based behavioral intervention for their young child with autism. *Journal of Autism and Developmental Disorders*.

[4] Karst J. S., van Hecke A. V. (2012). Parent and family impact of autism spectrum disorders: a review and proposed model for intervention evaluation. *Clinical Child and Family Psychology Review*.

[5] Osborne L. A., Reed P. (2010). Stress and self-perceived parenting behaviors of parents of children with autistic spectrum conditions. *Research in Autism Spectrum Disorders*.

[6] Davis N. O., Carter A. S. (2008). Parenting stress in mothers and fathers of toddlers with autism spectrum disorders: associations with child characteristics. *Journal of Autism and Developmental Disorders*.

[7] Zaidman-Zait A., Mirenda P., Duku E. (2014). Examination of bidirectional relationships between parent stress and two types of problem behavior in children with autism spectrum disorder. *Journal of Autism and Developmental Disorders*.

[8] Wachtel K., Carter A. S. (2008). Reaction to diagnosis and parenting styles among mothers of young children with ASDs. *Autism*.

[9] van Ijzendoorn M. H., Rutgers A. H., Bakermans-Kranenburg M. J. (2007). Parental sensitivity and attachment in children with autism spectrum disorder: comparison with children with mental retardation, with language delays, and with typical development. *Child Development*.

[10] Ainsworth M. D. S., Blehar M. C., Waters E., Wall S. (1978). *Patterns of Attachment: A Psychological Study of the Strange Situation*.

[11] Biringen Z., Robinson J. L., Emde R. N. (2000). Appendix B: the emotional availability scales (3rd ed.; an abridged infancy/early childhood version). *Attachment & Human Development*.

[12] Grusec J. E., Davidov M. (2010). Integrating different perspectives on socialization theory and research: a domain-specific approach. *Child Development*.

[13] Maccoby E. E., Martin J. A., Mussen P. H. (1983). Socialization in the context of the family: parent-child interaction. *Handbook of Child Psychology: Socialization, Personality, and Social Development*.

[14] Kelley S. A., Brownell C. A., Campbell S. B. (2000). Mastery motivation and self-evaluative affect in toddlers: longitudinal relations with maternal behavior. *Child Development*.

[15] Soenens B., Vansteenkiste M., Lens W. (2007). Conceptualizing parental autonomy support: adolescent perceptions of promotion of independence versus promotion of volitional functioning. *Developmental Psychology*.

[16] Kasari C., Sigman M., Mundy P., Yirmiya N. (1988). Caregiver interactions with autistic children. *Journal of Abnormal Child Psychology*.

[17] Watson L. R. (1998). Following the child's lead: mothers' interactions with children with autism. *Journal of Autism and Developmental Disorders*.

[18] Baker J. K., Messinger D. S., Lyons K. K., Grantz C. J. (2010). A pilot study of maternal sensitivity in the context of emergent autism. *Journal of Autism and Developmental Disorders*.

[19] Lemanek K. L., Stone W. L., Fishel P. T. (1993). Parent-child interactions in handicapped preschoolers: the relation between parent behaviors and compliance. *Journal of Clinical Child Psychology*.

[20] Wan M. W., Green J., Elsabbagh M., Johnson M., Charman T., Plummer F. (2012). Parent–infant interaction in infant siblings at risk of autism. *Research in Developmental Disabilities*.

[21] Meirsschaut M., Roeyers H., Warreyn P. (2011). The social interactive behaviour of young children with autism spectrum disorder and their mothers: is there an effect of familiarity of the interaction partner?. *Autism*.

[22] Blacher J., Baker B. L., Kaladjian A. (2013). Syndrome specificity and mother-child interactions: examining positive and negative parenting across contexts and time. *Journal of Autism and Developmental Disorders*.

[23] Rutgers A. H., van IJzendoorn M. H., Bakermans-Kranenburg M. J. (2007). Autism, attachment and parenting: a comparison of children with autism spectrum disorder, mental retardation, language disorder, and non-clinical children. *Journal of Abnormal Child Psychology*.

[24] Lambrechts G., Van Leeuwen K., Boonen H., Maes B., Noens I. (2011). Parenting behaviour among parents of children with autism spectrum disorder. *Research in Autism Spectrum Disorders*.

[25] Van Leeuwen K., Rousseau S., Hoppenbrouwers K., Wiersema R., Desoete A. (2011). *JOnG! Opvoedings—En Gezinsvariabelen bij de Vlaamse Geboortecohorte 0-Jarigen (Rapport 24)*.

[26] Van Leeuwen K. G., Vermulst A. A. (2004). Some psychometric properties of the ghent parental behavior scale. *European Journal of Psychological Assessment*.

[27] Van Leeuwen K., Noens I. (2013). *Parental Behavior Scale for Autism Spectrum Disorders*.

[28] Field A. (2005). *Discovering Statistics Using SPSS*.

[29] Cohen J. (1988). *Statistical Power Analysis for the Behavioural Sciences*.

[30] Cattell R. B. (1966). The scree test for the number of factors. *Multivariate Behavioral Research*.

[31] Maljaars J., Boonen H., Lambrechts G., Van Leeuwen K., Noens I. (2014). Maternal parenting behavior and child behavior problems in families of children and adolescents with autism spectrum disorder. *Journal of Autism and Developmental Disorders*.

[32] Berk L. E., Berk L. E. (1998). Emotional and social development in early childhood. *Development through the Lifespan*.

[33] Chawarska K., Volkmar F. R. (2005). Autism in infancy and early childhood. *Handbook of Autism and Pervasive Developmental Disorders*.

[34] Hobson P. R., Hobson J. A., Baron-Cohen S., Tager-Flusberg H., Lombardo M. V. (2013). Autism: self and others. *Understanding Other Minds: Perspectives from Developmental Social Neuroscience*.

[35] Hartley S. L., Sikora D. M., McCoy R. (2008). Prevalence and risk factors of maladaptive behaviour in young children with autistic disorder. *Journal of Intellectual Disability Research*.

[36] Charman T., Ricketts J., Dockrell J. E., Lindsay G., Palikara O. (2015). Emotional and behavioural problems in children with language impairments and children with autism spectrum disorders. *International Journal of Language and Communication Disorders*.

[37] Park C. J., Yelland G. W., Taffe J. R., Gray K. M. (2012). Brief report: the relationship between language skills, adaptive behavior, and emotional and behavior problems in pre-schoolers with autism. *Journal of Autism and Developmental Disorders*.

